# The effectiveness of telemedicine for paediatric retrieval consultations: rationale and study design for a pragmatic multicentre randomised controlled trial

**DOI:** 10.1186/s12913-014-0546-9

**Published:** 2014-11-11

**Authors:** Nigel R Armfield, Mark G Coulthard, Anthony Slater, Julie McEniery, Mark Elcock, Robert S Ware, Paul A Scuffham, Mark E Bensink, Anthony C Smith

**Affiliations:** Centre for Online Health, School of Medicine, The University of Queensland, Brisbane, Australia; Queensland Children’s Medical Research Institute, The University of Queensland, Brisbane, Australia; Academic Discipline of Paediatrics and Child Health, School of Medicine, The University of Queensland, Brisbane, Australia; Paediatric Intensive Care Unit, Royal Children’s Hospital, Brisbane, Australia; Retrieval Services Queensland, Department of Health, Brisbane, Australia; School of Population Health, The University of Queensland, Brisbane, Australia; Griffith Health Institute, School of Medicine, Griffith University, Brisbane, Australia; Centre for Online Health, Royal Children’s Hospital, Level 3, Foundation Building, Herston, Queensland 4029 Australia

**Keywords:** Paediatrics, Paediatric intensive care, Paediatric critical care, Telemedicine, Transport

## Abstract

**Background:**

In many health systems, specialist services for critically ill children are typically regionalised or centralised. Studies have shown that high-risk paediatric patients have improved survival when managed in specialist centres and that volume of cases is a predictor of care quality. In acute cases where distance and time impede access to specialist care, clinical advice may be provided remotely by telephone. Emergency retrieval services, attended by medical and nursing staff may be used to transport patients to specialist centres. Even with the best quality retrieval services, stabilisation of the patient and transport logistics may delay evacuation to definitive care. Several studies have examined the use of telemedicine for providing specialist consultations for critically ill children. However, no studies have yet formally examined the clinical effectiveness and economic implications of using telemedicine in the context of paediatric patient retrieval.

**Methods/Design:**

The study is a pragmatic, multicentre randomised controlled trial running over 24 months which will compare the use of telemedicine with the use of the telephone for paediatric retrieval consultations between four referring hospitals and a tertiary paediatric intensive care unit. We aim to recruit 160 children for whom a specialist retrieval consultation is required. The primary outcome measure is stabilisation time (time spent on site at the referring hospital by the retrieval team) adjusted for initial risk. Secondary outcome measures are change in patient’s physiological status (repeated measure, two time points) scored using the Children’s Emergency Warning Tool; change in diagnosis (repeated measure taken at three time points); change in destination of retrieved patients at the tertiary hospital (general ward or paediatric intensive care unit); retrieval decision, and length of stay in the Paediatric Intensive Care Unit for retrieved patients. The trial has been approved by the Human Research Ethics Committees of Children’s Health Services Queensland and The University of Queensland, Australia.

**Discussion:**

Health services are adopting telemedicine, however formal evidence to support its use in paediatric acute care is limited. Generalisable evidence is required to inform clinical use and health system policy relating to the effectiveness and economic implications of the use in telemedicine in paediatric retrieval.

**Trial registration:**

Australian and New Zealand Clinical Trials Registry ACTRN12612000156886.

## Background

In many health systems, specialist health services for critically ill children are organised regionally, typically in areas of high population. In this model, larger centres provide paediatric intensive care services and have ready access to a range of sub-speciality expertise. These specialist centres may also provide referring facilities with clinical advice by telephone and also contribute to, or be supported by, specialised paediatric patient retrieval services.

The clinical argument favouring regionalisation is that the concentration of expertise and resources, high patient volume and co-ordination of services within a geographic area leads to improved patient outcomes. [1] While a relationship between volume and outcome for children managed in paediatric intensive care units (PICU) has not been clearly established [2,3], studies have shown that high-risk paediatric patients have improved survival when managed in tertiary centres [4] and that volume of cases is positively associated with care quality [3]. It has also been shown that the centralisation of paediatric intensive care services, in combination with a specialised patient retrieval service, has clinical benefits [5]. There is also an economic argument for regionalisation: geographical factors and population distribution may dictate that regionalisation is the only affordable ways for a health system to provide complex specialist care. However, while the regionalised model may be both clinically beneficial and in some cases economically necessary, in urgent and emergent situations distance and time impediments may disadvantage those who live outside of the major centres of population.

When a critically ill child presents at a referring centre, the quality and timeliness of communication with clinicians at the specialist centre is very important. In this context, real-time telemedicine based consultations, alongside high-quality local care and a well-coordinated retrieval service, may have a useful role. It may be that consultation by telemedicine has advantages over use of the telephone arising from the ability to view clinically useful visual information, including directly observing the child, procedures, medical images and equipment such as the patient monitor output and ventilator settings.

The use of telemedicine is not new [6] and its potential role in paediatric critical care was first discussed by Wetzel in 2001 [7] at a time when the use of telemedicine in adult intensive care units (ICU) was still embryonic and PICU applications had yet to be developed. Subsequently, early studies of telemedicine for managing seriously ill children first began to appear in the literature in 2004, with the majority of the reported work being led by investigators at The University of California Davis (UCD). The first reported study examined the use of telemedicine to support the care of children admitted as inpatients in a remote adult ICU in Northern California [8]. This two year study, involving 70 consultations for 47 patients, found that children who received telemedicine consultations from a PICU at the UCD Medical Center (UCDMC) had mortality and length of stay (LoS) outcomes that were comparable to those of severity-adjusted reference benchmark data from 33 national PICUs. The study also reported that the referring clinicians and parents had high satisfaction with the use of telemedicine. Overall, the study suggested that it was feasible to provide quality care to some children in the community hence avoiding transport and inconvenience for families. The investigators also conducted an economic analysis [9] which reported annual hospital and transport savings of USD172,000 for patients who received telemedicine consultations and USD300,000 for patients who received telemedicine consultations and for whom transport was determined to be avoided. By retaining patients in the adult ICU, the referring hospital also increased their annual revenue by USD186,000 for patients who received telemedicine consultations and USD279,000 for patients for the patients who received telemedicine consultations and for whom transport was avoided. The annual cost of providing telemedicine was reported as USD120,000 economic benefit.

Also in 2004, Marcin et al. reported a second study of telemedicine consultation. [10] In this study 17 paediatric trauma patients who had been admitted to the same remote adult ICU were recruited. Telemedicine consultations were provided from UCDMC at the discretion of the referring clinician. This study also concluded that the approach was feasible for trauma care and was considered satisfactory by the referring clinicians and families. In 2005, Kon and Marcin reported two case studies of the use of telemedicine to support paediatric resuscitation [11]. In these examples, an intensivist observed and provided advice by telemedicine from home. While in one case resuscitation was ultimately unsuccessful, the attending teams reported that telemedicine had benefits because the intensivists could directly observe cardio-pulmonary resuscitation, bagging and the patient monitor and recommend interventions based on visual information that would not be available during a telephone call.

Between 2006 and 2008, Health et al. conducted a two year prospective study of intensivist telemedicine consultations to rural emergency departments (ED). [12] They reported the results of 63 consultations conducted across 10 rural EDs. Six point questionnaires were used to survey clinician opinions of telemedicine. The majority of both referring and providing clinicians agreed, or strongly agreed (referring 87.5%, providing 88.9%), that telemedicine improved the quality of care for the patient. Referring and providing clinicians differed in their views of whether the consultation could have been conducted as well by telephone, with 55% of referring clinicians disagreeing, or strongly disagreeing with the statement, compared with 90.5% of providing clinicians. Opinions on the technical quality of the consultations and on the quality of clinician communications were comparable between referring and providing clinicians, with the majority agreeing or strongly agreeing that the quality was high.

In 2012, Yager at al. examined the use of telemedicine for providing night-time consultations by intensivists to a PICU from home. [13] This descriptive retrospective study reviewed the audio and video quality, duration, reason for consultation, persons present and changes in medical management for 56 out-of-hours consultations. The study concluded that the use of telemedicine was feasible and valuable, though the effect on patient outcomes was not assessed.

In 2013 using a retrospective chart review, LaBarbera et al. identified that telemedicine consultations between intensivists and a community hospital physicians, together with the implementation of a paediatric hospitalist program at the community hospital had the potential to reduce the need for patient transport to the PICU. [14] Also using retrospective chart review, a validated implicit quality review tool and surveys, Dharmar et al. compared the quality of care provided to children who received a telemedicine consultation with a paediatric intensivist, a telephone consultation with a paediatric intensivist or no paediatric intensivist consultation at all. The study found that patients who received telemedicine consultations had the highest quality of physician-reported quality of care, intermediate quality was reported for telephone consultations and the lowest quality was reported for no intensivist consultation. [15] Dharmar et al. also compared medication errors in groups of children who received telemedicine consultations, telephone consultations or no consultation. The children who received telemedicine consultations experienced significantly fewer physician-related medication errors (3.4%) compared with those who received telephone consultations (10.8%) or no consultations at all (12.5%) [16].

In general, while telemedicine is not new, the formal evidence to support its use remains weak. [17] While pragmatic randomised controls may are a useful method of producing evidence for clinicians and policy-makers, very few have been published in telemedicine. In paediatric critical care specifically, there is very limited high-quality evidence. In their 2012 systematic review and meta-analysis, Wilcox et al. [18] identified no randomised studies of telemedicine in critical care. In their pooled analysis of 9 observational studies they found that the use telemedicine was associated with a reduction in ICU and hospital mortality (RR 0.79; 95% CI 0.65 to 0.96 and RR 0.83 95% CI 0.73 to 0.94 respectively). However, the pooled analysis included only one study of paediatric patients (Marcin et al. [8]) and that study favoured the control over telemedicine and had a wide confidence interval (RR 1.06, 95% CI 0.13 to 8.87). Wilcox also found that telemedicine was associated with a statistically significant reduction in LoS (both in ICU and in hospital) however no studies involving paediatric patients were reported in the pooled estimates.

While observational studies suggest that telemedicine may have clinical and economic benefits when compared with consultation by telephone, formal evidence is needed.

The aim of this study is to formally explore the clinical and economic effects of providing telemedicine consultations from a PICU to referring hospitals in Queensland’s regionalised public health care system.

### Objectives and hypotheses

#### Hypotheses

Consultation using real-time video-based telemedicine between a PICU consultant and a referring hospital clinician will:

- Clinical effectiveness
  1. Reduce the time needed by the retrieval team to stabilise the child before transport
  2. Improve the child’s condition between time of initial call and time of retrieval team arrival
  3. Reduce diagnostic discordance
  4. Reduce the number of retrieved children being admitted to general wards at the tertiary hospital
  5. Reduce PICU LoS for retrieved children
- Economic effects
  1. From the health service perspective, be economically beneficial by reducing the number, and consequently the cost, of (i) retrievals, (ii) unnecessary tertiary hospital admissions, and (iii) LoS.

## Methods/Design

### Design

This study is a pragmatic four-centre open randomised controlled trial. Patients recruited to the intervention arm will receive a telemedicine consultation, while patients recruited to the control arm will receive a telephone consultation (usual care). The study will be conducted over 24 months.

This study is focussed on the role of telemedicine in the management paediatric retrievals and hence will randomise retrieval calls only, and have a retrieval-related primary outcome measure. However, information will also be collected for telemedicine-based clinical advice-only calls which are placed directly to the PICU and this information will be used for separate secondary observational analyses.

The study has been approved by the Queensland Children’s Health Services (RCH) Human Research Ethics Committee (HREC/11/QRCH/175) and The University of Queensland Medical Research Ethics Committee (2012000136). The study will be conducted in accordance with the principles of the Declaration of Helsinki and reported in accordance with the CONSORT statement.

### Setting

Queensland is Australia’s second largest state/territory with an area of over 1.7 million square kilometres. It has a population of over 4.5 million, has around 60,000 births per year and high population growth in regional areas. However, Queensland’s two tertiary children’s hospitals are both located in the state capital of Brisbane in the southeast corner of the state, in effect centralising speciality and sub-speciality care.

The Royal Children’s Hospital (RCH) PICU provides a state wide advice and retrieval service. Approximately 230 paediatric patients who need critical care are retrieved to the unit each year. Clinical advice and retrieval management between the PICU and referring hospitals is provided by telephone. First point of call, clinical co-ordination, prioritisation and tasking of resources for all paediatric retrievals is conducted centrally by Retrieval Services Queensland (RSQ) based at the Queensland Emergency Medical System Coordination Centre in Brisbane.

### Study site selection

The study is funded to provide the RCH PICU and four study sites with telemedicine equipment. To maximise recruitment within the resource constraints of the project, retrospective data were examined to identify four referring hospitals which consistently contributed to the PICU retrieval workload over two recent years (2010, 2011). For this period, when referring hospitals were ranked by retrieval activity, the top four hospitals accounted for 220 patients (i.e. approximately 110 patients per year). These cases represent almost half of the total retrieval workload of the PICU. These study sites are all large regional public hospitals, each with an emergency department, paediatric ward and paediatricians on staff. In some cases, adult ICUs at the referring hospitals may admit paediatric patients if needed. Site round trip distances from the RCH PICU range from 62 km to 1042 km (Table 1). The four sites identified will be provided with telemedicine links to the RCH PICU.

**Table 1:** **Study sites, usual retrieval mode and round trip distance from the RCH**

**Study site**	**Usual mode of retrieval**	**Round trip distance**	
		**Air (nm; km)**	**Road (km)** ^**1,3**^
Redcliffe Hospital	Road ambulance	N/A^2^	62
Nambour General Hospital	Helicopter	100;185	N/A^2^
Bundaberg Base Hospital	Fixed wing aircraft	312;578	41
Rockhampton Base Hospital	Fixed-wing aircraft	562;1042	36.4

^1^Using main roads; ^2^Direct hospital-to-hospital; ^3^Fixed wing retrievals include road-ambulance components between the referring hospital and local airport, and between the Royal Children’s Hospital and the Royal Flying Doctor Service at Brisbane Airport.

### Participants

#### Patients

Study participants will be male or female children, aged 15 years or under, who are inpatients, or who present at the emergency departments of any of the four regional referring study hospitals and for whom a consultation with the tertiary PICU is required. Reasons for such consultations may be to seek clinical advice from the PICU, and/or to plan retrieval of the child. Informed consent will be obtained from a parent or guardian by a clinician at the referring hospital before recruitment. A patient information sheet and consent form will be provided for this purpose.

#### Clinicians

Because the video and audio of telemedicine consultations will be recorded for retrospective analysis, informed consent will also be required from participating clinicians at both the PICU and regional referring hospitals. This process will be conducted by the investigators at commencement of the trial.

### Randomisation

Generation of intervention/control arm allocation sequences will occur centrally. Sequences will be generated using permuted block randomisation, with random block sizes of 6,8 or 10 entries and a 1:1 allocation ratio using an online true random number generation service [19]. The process of generating these sequences will be conducted by an independent party. Four tables of random numbers will be generated, one for each study site. Odd numbers will denote allocation to the control arm and even numbers to the intervention. The tables will be held securely on a server on a private network.

A password-protected custom application (accessible by computer or portable device), driven by the random number tables, will be provided for the PICU clinicians to determine cases and controls at time of initial retrieval call from RSQ. The application will record all accesses and allocations.

### Masking

It is not possible to conceal study arm allocation from patients, clinicians or investigators. However data extractors will be masked, except when extracting audio and video related information which will be collected for the intervention arm only.

### Patient recruitment

Patients will be recruited from the referring hospital paediatric wards, emergency departments or adult intensive care units.

**Figure 1:**
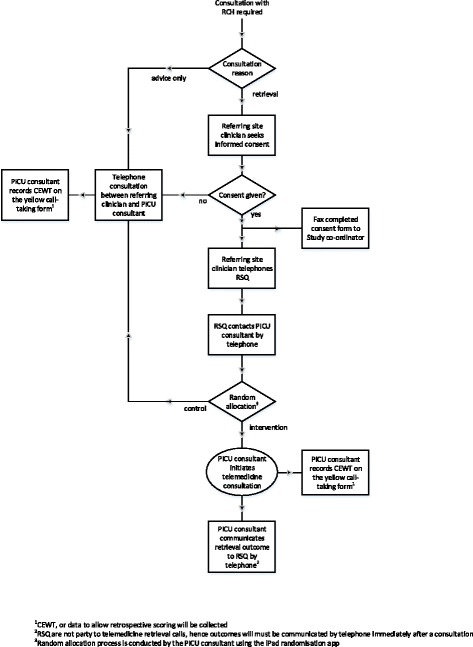
**Recruitment flowchart.**

Initial contact between the referring hospital clinician and RSQ will always be made by telephone (Figure 1). On receiving that call, the RSQ nurse co-ordinators, will then also contact the designated PICU consultant by telephone. Using the randomisation process, the PICU consultant will determine whether the consultation continues by telephone (usual care, control arm), or by telemedicine (intervention arm). While all parties will aim to comply with the study protocol, there may be circumstances under which the referring and PICU clinicians elect to override the randomisation process in the interest of the care of the child. The resulting loss of study cases will be monitored and recorded on a daily basis.

#### Intervention arm-telemedicine

Patients randomised to the intervention arm will receive a consultation by real-time audio-visual telemedicine. This form of consultation allows clinicians to see and hear each other at a distance. It also allows the specialist at the PICU to remotely view the patient, medical images and medical equipment (e.g. the patient monitor and/or ventilator). Visual information will be used by the specialist when providing clinical advice, observing interventions and in managing retrievals. This form of telemedicine allows the clinician at the patient end to work hands free.

Custom designed telemedicine systems and associated infrastructure [20] will be installed at each site. The efficacy of these systems has been extensively tested in the controlled environments of a neonatal intensive care unit [21,22] and a PICU [23,24]. In some circumstances, consultations may also be conducted using existing health department video conferencing systems.

#### Control arm-usual care

Patients randomised to the control arm will receive a telephone consultation. During the consultation, clinical information such as the patient’s condition, descriptions of medical images and output/settings on medical equipment will be conveyed verbally. No visual information will be shared between hospitals. Telephone consultations of this style are the current usual practice in all four participating centres.

#### Advice-only calls

Advice-only calls will not be randomised and clinicians may elect to use telephone, or to use telemedicine, or possibly a combination of both. These calls will not be co-ordinated by RSQ, rather the referring hospital clinician will contact a clinician at the RCH PICU directly. These patients will not be recruited to the RCT, but will be recruited to a separate observational study.

### Outcome measures

**Figure 2:**
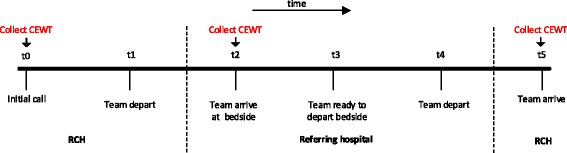
**Retrieval process time points.**

Time points (*t0*–*t5*) in the retrieval process are shown in Figure 2.

#### Primary outcome measure

1. Stabilisation time (*ST*). Clinical time spent by the retrieval team with at the bedside at the referring hospital (*ST* = *time*_*t*3_ − *time*_*t*2_). We will analyse all participants after stratifying for initial risk, as measured using the Paediatric Index of Mortality 3 (PIM3) [14].

#### Secondary outcome measures

1. Child’s physiological status using repeated measure scored using the Children’s Emergency Warning Tool (CEWT) (Authors: Royal Children’s Hospital, Queensland Health, unpublished) at two time points: (1) at time of initial call from the referring hospital (*CEWT*_*t*0_); and (2) at time of arrival of the retrieving team at the bedside at the referring hospital (*CEWT*_*t*2_).
  CEWT is an observation-chart based tool used within hospitals in Queensland. The tool is designed to identify patient deterioration and to trigger appropriate clinical escalations. The tool has nine components: respiratory rate, respiratory distress, Oxygen rate, Oxygen saturation, temperature, heart rate, blood pressure, capillary refill time, and level of consciousness. Observations are charted at the bedside, as with a conventional patient chart. Within each component, observations are translated to a score, on a scale of 1 (best) to 3 (worst). A score of ‘E’ and purple colour coding is used to prompt an emergency response (e.g. a respiratory rate <10 breaths/min, heart rate <60 beats/min or any component of blood pressure <50 mmHg).
  After each patient assessment, a total CEWT score is calculated by summing the component scores. A range of clinical responses are triggered by both significant negative changes in between-assessment component scores, and by the total CEWT score.
2. Change in diagnosis (*DX*) using repeated measure taken at three time points: (1) at time of initial call to RSQ (*DX*_*t*0_); (2) at time of arrival of the retrieval team at the bedside at the referring hospital (*DX*_*t*2_); and (3) at time of arrival of the retrieval team and retrieved patient back at the PICU (*DX*_*t*5_).
3. Destination of retrieved patients at the tertiary hospital (i.e. general ward vs. PICU).
4. Retrieval decision (retrieval conducted/not conducted)
5. PICU LoS for retrieved patients.

### Data collection

This study will use routinely collected patient care and health service reporting data. Sources will be: (1) the retrieval form, for clinical and timing information relating to the retrieval mission; (2) the patient chart, for diagnosis information and admission location at the tertiary hospital. For cases in the intervention arm, the audio and video of all consultations will be recorded to allow retrospective analysis. An activity log will be maintained to provide summary statistics of telemedicine and telephone usage during the study period.

Economic data routinely collected will include hospital admission data, length of stay by ward type, procedures, and Diagnostic Related Groups (DRGs). Additional data to be collected during the trial will include staffing, resource use and costs for the intervention, and the distance travelled by patients and their families. References for economic analyses will be: (1) The National Hospital Cost Data Collection Cost Report Round 14 (2009–2010) [25]; (2) current transport costs (sourced from emergency transport providers) and current (3) Queensland Health salary scales for staff costs.

### Sample size

In this study, resources limit the number of study sites to four and the study duration to 24 months. It is estimated that during the study period there will be 200 retrieval calls relating to eligible participants. We estimate an 80% participation rate (20% attrition due to lack of consent or clinicians electing not to consult by telemedicine and continuing by telephone) and consequently estimate the study will recruit a total of 160 retrieval participants with 80 in each arm. Retrospective stabilisation time data for the study sites showed a standard deviation of 41 minutes. With alpha = 0.05 we have 80% power to detect a clinically important difference in stabilisation time between-consultation types of 18 minutes or greater.

### Data analysis

Data analysis will be conducted under the intention-to-treat (ITT) principle. Descriptive data will be presented as either mean (standard deviation) or median (25th to 75th percentile) for continuous data depending on its distribution, and as frequency (percentage) for categorical data. Statistical analyses for each outcome variable are shown in Table 2. All statistical analyses will be conducted using Stata (Statacorp, College Station).

**Table 2:** **Statistical analyses for each outcome measure**

**Outcome measure**		**Statistical Method**
P1	Stabilisation time^1^	Linear regression with stabilisation time as the outcome, type of consultation as the main effect and PIM3 score as a co-variable
S1	Change in patient’s physiological status	Linear mixed effects model with physiological status as outcome, type of consultation and time as main effects, and a consultation-by-time interaction term.
S2	Change in diagnosis	Logistic mixed effects model with diagnosis as outcome, type of consultation and time as main effects, and a consultation-by-time interaction term.
S3	Destination of retrieved patients	Logistic regression with destination as outcome and type of consultation as the main effect.
S4	Retrieval decision	Logistic regression with retrieval decision as outcome and type of consultation as the main effect.
S5	PICU LoS^1^	Linear regression with LoS as the outcome, and type of consultation as the main effect.

^1^Data relating to stabilisation time and PICU LoS will transformed if necessary to meet assumptions of regression model.

### Economic analysis

A cost-minimisation analysis will be undertaken. Resource use collected during the trial will be costed with unit costs applied to each resource to derive the cost per case. Costs per day stay in PICU and other hospital wards will be estimated on a *per diem* basis to more accurately estimate total hospital costs for this group of patients (PICU costs are typically skewed due to some cases having very long stays with exceptionally high costs).

All cost data will be standardised to current values. No discounting will be necessary due to the short time horizon (<12 months) for the analysis. The group with the lowest costs will be deemed as the optimal strategy. One way deterministic sensitivity analysis will be undertaken to identify factors affecting the stability of the results.

## Discussion

As specialist care becomes more centralised and the cost of providing care increases, telemedicine may be a way to improve outcomes in a cost-effective way. However, little is known about the use of telemedicine in the complex, risky and expensive area of paediatric acute care retrieval.

Using a pragmatic randomised design, with telemedicine consultation as the intervention and telephone consultation as the control, this study will provide new information on the effectiveness and economics of using telemedicine for paediatric acute care retrieval management in a regionalised public health care system. In particular, it will determine whether using telemedicine for specialist consultation prior to patient retrieval results in a more stable patient and hence lead to their more efficient evacuation to definitive care. It will also examine whether the use of telemedicine improves diagnosis, can improve the appropriateness of retrieval and reduce the length of stay in PICU. The study incorporates an economic analysis to allow the observed effects of telemedicine to be costed.

The results of the study will be useful to both clinicians and health service policy makers in the Australian context and in other regionalised or centralised specialist health care systems.

## Abbreviations

CEWT: Children’s Early Warning Tool
DRG: Diagnostic Related Group
ED: Emergency Department
ICU: Intensive Care Unit
ITT: Intention to Treat
LoS: Length of Stay
RCH: Royal Children’s Hospital
PICU: Paediatric Intensive Care Unit
PIM3: Paediatric Index of Mortality 3
RCT: Randomised Controlled Trial
RSQ: Retrieval Services Queensland
UCD: University of California Davis
UCDMC: University of California Davis Medical Center
USD: United States Dollars
